# Left main coronary artery atresia with concomitant mitral regurgitation in an adult: A case report

**DOI:** 10.1097/MD.0000000000012367

**Published:** 2018-10-12

**Authors:** Meice Tian, Xianqiang Wang, Huawei Gao, Liqing Wang, Shengshou Hu

**Affiliations:** Department of Surgery, National Center for Cardiovascular Disease, Fuwai Hospital, Chinese Academy of Medical Sciences & Peking Union Medical College, Beijing, China.

**Keywords:** coronary artery bypass graft, left main coronary artery atresia, mitral regurgitation

## Abstract

**Introduction:**

Left main coronary artery (LMCA) atresia is a quite rare congenital malformation, which may present with various symptoms. Past literatures were sporadic without recent summary of world-wide cases. We hereby report an adult case of LMCA atresia with concomitant mitral regurgitation and also summarize all cases found in published literatures.

**Case presentation:**

A 48-year old female presented with sudden dyspnea. Preliminary impression was acute heart failure caused by mitral regurgitation. Preoperative coronary angiography demonstrated that there was no left coronary ostium and multiple collateral vessels arising from right coronary artery. The diagnosis of left main coronary atresia was made and the patient received successful valvuloplasty and coronary artery bypass grafting with left internal mammary artery anastomosed to the left anterior descending (LAD) artery. She recovered well and 3-month follow-up showed the graft was patent.

**Conclusion:**

This case highlights the importance of angiography for diagnosis of LMCA and performance of CABG once diagnosed.

## 1 Introduction

Left main coronary artery (LMCA) atresia is a rare congenital malformation, which is characterized by absence of left coronary ostium and left main trunk in the left coronary artery system. Patients may be asymptomatic or present with syncope, angina pectoris, myocardial infarction, or sudden death.^[1]^ Diagnosis relies on coronary angiography, particularly to distinguish from single coronary artery or abnormal origin. Since 1955, there have been sporadic reports of LMCA atresia, however with no reports of this abnormality in recent years.^[2]^ Hereby, we report a case from our own institution, who was first admitted for mitral regurgitation and then detected LMCA atresia by preoperative catheterization and computed tomography (CT) coronary angiography. The patient successfully underwent mitral valve repair and concomitant coronary artery bypass grafting (CABG) surgery with the left internal thoracic artery (ITA) anastomosed to the left anterior descending artery (LAD). Besides, we also reviewed all literatures reporting this disease so far, and comprehensively summarized the characteristics of all cases found in literatures. Informed consent was obtained from the patient for publication of this case report and accompanying images.

## 2 Case report

A 48-year-old female was sent to the emergency department of our hospital with the chief complaint of sudden occurrence of dyspnea. The patient experienced sudden respiratory distress when she was lifting heavy cargo during farm work. She also complained orthopnea with pink bubble sputum cough. The patient was 155 cm in height and weighed 48 kg. Her heart rate was 103 per minute, and blood pressure was 99/63 mm Hg. Physical examination revealed a 4/6 systolic murmur in cardiac apex. Electrocardiogram demonstrated depression of ST segments at multiple leads: I, II, aVL, and V3 to V6. Meanwhile, blood test showed no troponin elevation. Acute pulmonary congestion was detected by chest X-ray. Echocardiogram found a prolapsed posterior leaflet of the mitral valve with massive regurgitation, with an ejection fraction (EF) of 68% and a normal sized left ventricle. The patient denied of similar symptoms in her past history.

**Figure 1:**
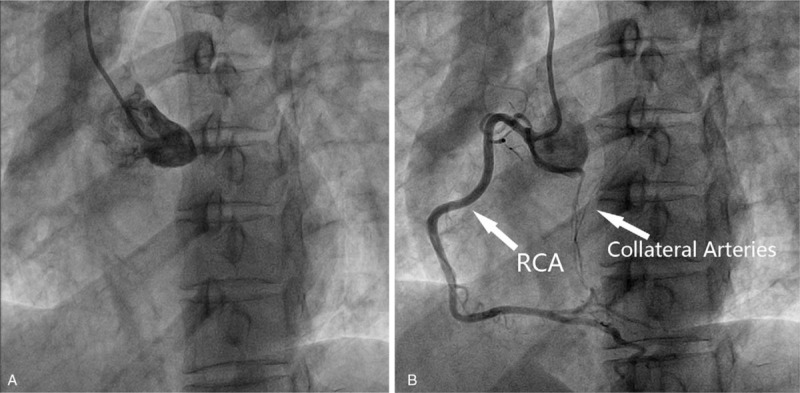
Preoperative coronary angiography showing that (A) the left coronary ostium could not be catheterized, indicating it was absent; (B) multiple collateral arteries flowing from the right coronary artery to the left coronary area. RCA = right coronary artery.

Therefore, she was preliminarily diagnosed of acute left heart failure caused by mitral regurgitation and scheduled for valve repair surgery. Routine coronary angiography was performed to exclude coexisting coronary artery disease. However, the left main coronary trunk could not be catheterized. Meanwhile, collateral vessels could be seen flowing from right coronary artery (RCA) to fill the left coronary system (Fig. 1). The patient was then suspected of left main coronary atresia or complete occlusion. She received CT coronary angiography for further diagnosis, which confirmed absence of left coronary ostium, with abnormally small LAD and left circumflex arteries (LCX), both filled by collateral vessels arising from RCA.

**Figure 2:**
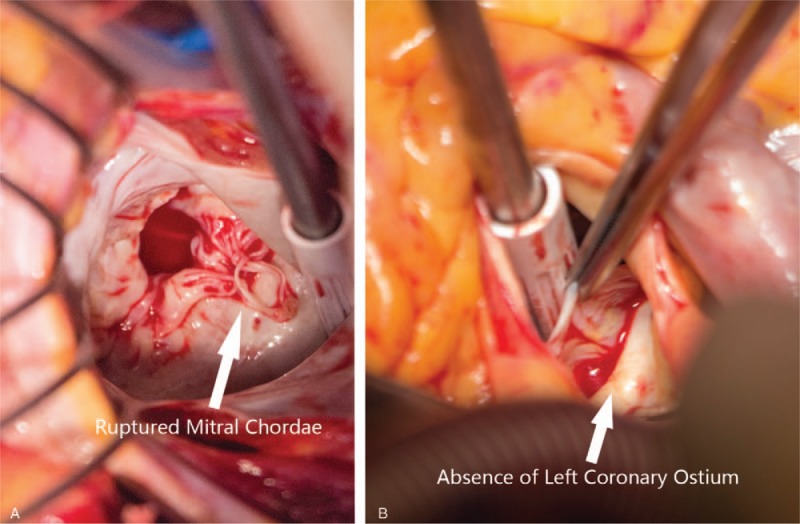
Findings by direct inspection during operation, showing that (A) ruptured chordae of the posterior mitral leaflet; (B) no left main coronary artery trunk nor left aortic sinus could be identified through aortotomy.

During operation, the patient's mitral chordae of the posterior leaflet was found ruptured, with dysplasia of papillary muscle (Fig. 2). Then, a successful mitral valvuloplasty was first performed. Although with worry that the small caliber left coronary system may not be a suitable target for surgical revascularization, we performed CABG with left internal mammary artery anastomosed to proximal LAD anyway. Graft flow was satisfactory, with a flow value of 28 mL/min and pulsatile index of 1.2. By direct inspection through aortotomy, no LMCA trunk nor left aortic sinus could be identified. Therefore, the diagnosis of left main coronary atresia was confirmed.

**Figure 3:**
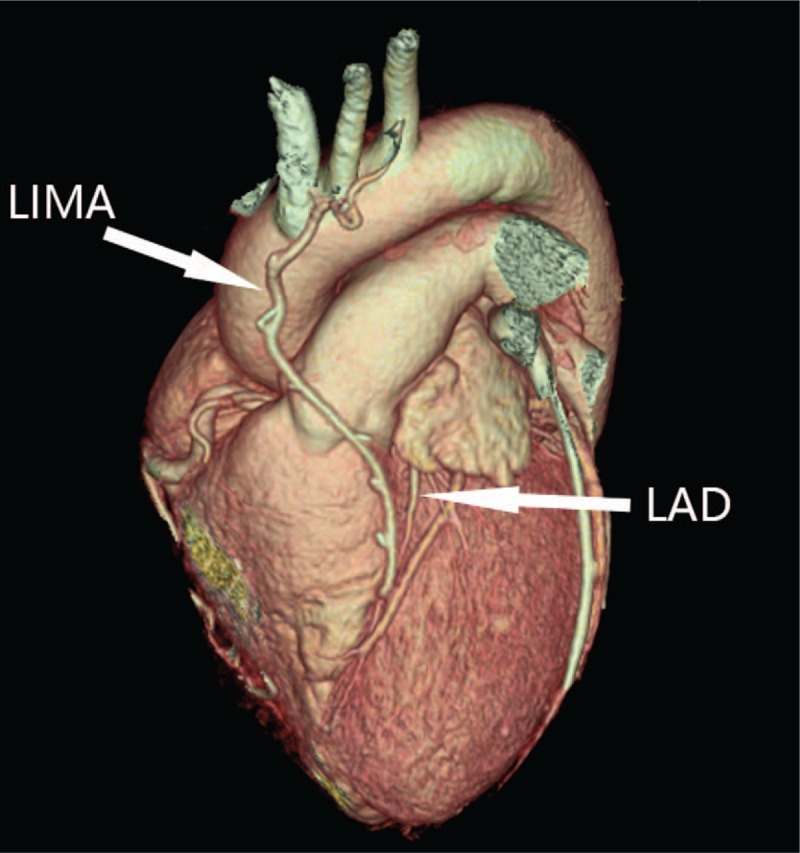
Three-month follow-up CT coronary angiography showed patent LIMA-LAD graft with the small LAD. CT = computed tomography, LAD = left anterior descending, LIMA = left internal mammary artery.

The patient recovered uneventfully and discharged 7 days after operation. Three months later, she came back for follow-up visit and received routine examination together with CT angiography. Her heart function was normal as revealed by echocardiography, and coronary angiography showed a patent LIMA-LAD graft with a small LAD (Fig. 3).

## 3 Discussion

LMCA atresia is a quite rare condition, with unclear etiology. A systemic or infectious (syphilitic) cause has been suggested for congenital and even acquired coronary ostium stenosis or atresia.^[3–5]^ Koh et al^[6]^ reported etiologies, including congenital absence of one coronary anlage, failure of development or displacement of one coronary anlage, failure of canalization of the proximal segment of the left coronary artery, involvement of the left coronary ostium due to fibrotic change in the aortic media, and coronary obstruction by infection or thrombosis in the early embryonic stage. The diagnosis of LMCA atresia relies on angiographic findings, which usually show absence of left coronary ostium, and left coronary artery filled in a retrograde manner via the RCA instead of antegrade blood flow. Anomalous origin of the left coronary from the pulmonary artery is one situation that should be differentiated with, which can be ruled out by coronary angiography.

To the best of our knowledge, from February 1955 to August 2017, a total of 70 cases have been reported according to published literatures, of whom 37 were children or adolescents (younger than 18 years) and 28 were adults, with 5 unknown of age. There were 35 males and 25 females, and in 10 cases, the gender was not specified.

In true LMCA atresia, left coronary system receives blood only by collateral arteries from the RCA, such as conal artery, intraseptal artery, apical artery, and so on. However, the heart may eventually be unable to cope with collateral circulations and develop myocardial ischemia.^[5]^ Of these 70 patients, 63 (90.0%) were symptomatic. Infants mostly present with failure to thrive and myocardial infarction.^[7–9]^ Children and adolescents often have syncope and tachyarrhythmia.^[10]^ Adult patients usually become symptomatic (angina pectoris) at an advanced age—when collateral flow cannot keep pace with myocardial demands.^[5]^

Concomitant abnormalities were not very common, which were found in 20 cases (28.6%). Among these, 8 were diagnosed with coexisting mitral regurgitation^[5,11–17]^ and 2 with aortic or supravalvular aortic stenosis.^[18–20]^ Interestingly, none of these 70 cases were found with concomitant atherosclerosis. Hung^[21]^ reported that the main mechanism of ischemic mitral regurgitation relates to the distortion of spatial relationships between the mitral valve and the papillary muscles secondary to ventricular remodeling. In our case, the patient did suffer from dysplastic mitral papillary muscle and ruptured chordae, caused by insufficient perfusion of left ventricle due to absence of LMCA. However, it is still hard to accurately define the relationship between LMCA atresia and mitral regurgitation, as diagnosed cases with both lesions are limited.

Prognosis of LMCA atresia is unfavorable. As suggested by previous literatures, surgical revascularization is probably the treatment of choice for both symptomatic and asymptomatic patients. In this regards, CABG using left mammary artery to the left coronary system seems to be the choice for adult patients, although long-term results in children remain questionable.^[11,19,22]^ Concerns were raised that the small caliber left coronary vessels may not be suitable targets for revascularization; however, some scholars suggested that once sufficient blood flow is established, these vessels grow in diameter and collateral arteries tend to disappear.^[22,23]^ As we reviewed, a total of 13 of the 27 (48.1%) adult patients received CABG, with no in-hospital death. Twelve of the 37 (32.4%) young cases underwent CABG, and 10 (27.0%) children received successful angioplasty to reconstruct left coronary ostium with autologous pericardium. Of the 70 cases, 3 children died postoperatively, of whom 1 received angioplasty and died of low cardiac output syndrome,^[24]^ and another 2 cases died due to failure of surgical left coronary reconstruction.^[7,25]^ Although long-term outcomes of the surviving patients were unknown, it is generally proposed that surgical intervention is the optimal choice. Our own patient also received successful CABG and 3-month follow-up demonstrated good recovery with patent LIMA-LAD graft. Continuous observation for longer-term efficacy is warranted.

In conclusion, LMCA atresia is a rare condition with varying symptoms. Its diagnosis relies on coronary angiography and attention shall be paid to concomitant anomalies. Once diagnosed, surgical treatment is probably favorable to restore myocardial blood flow for both symptomatic and asymptomatic patients.

## Author contributions

**Conceptualization:** Meice Tian, Xianqiang Wang, Huawei Gao, Liqing Wang, Shengshou Hu.

**Data curation:** Meice Tian.

**Investigation:** Meice Tian.

**Methodology:** Meice Tian, Xianqiang Wang, Huawei Gao, Shengshou Hu.

**Project administration:** Shengshou Hu.

**Supervision:** Liqing Wang.

**Writing – original draft:** Meice Tian.

## References

[1] Musiani A Cernigliaro C Sansa M Left main coronary artery atresia: literature review and therapeutical considerations. Eur J Cardiothorac Surg 1997;11:505–14. 9105816 10.1016/s1010-7940(96)01121-9

[2] Goormaghtigh N De Vos Blanquaert A Ostial stenosis of the coronary arteries in a nine years old girl. Arch Intern Med 1955;95:341–8. 10.1001/archinte.1955.00250080163017 13227658

[3] Goormaghtigh N De Vos L Blancquaert A Ostial stenosis of coronary arteries in nine-year-old girl. AMA Arch Intern Med 1955;95:341–8. 13227658 10.1001/archinte.1955.00250080163017

[4] Levin DC Fellows KE Abrams HL Hemodynamically significant primary anomalies of the coronary arteries. Angiographic aspects. Circulation 1978;58:25–34. 348342 10.1161/01.cir.58.1.25

[5] Elian D Hegesh J Agranat O Left main coronary artery atresia: extremely rare coronary anomaly in an asymptomatic adult and an adolescent soccer player. Cardiol Rev 2003;11:160–2. 12705847 10.1097/01.CRD.0000064423.27902.BB

[6] Koh E Nakagawa M Hamaoka K Congenital atresia of the left coronary ostium: diagnosis and surgical treatment. Pediatr Cardiol 1989;10:159–62. 2798191 10.1007/BF02081680

[7] Verney RN Mnnet P Arnaud P Myocardial infarction in an infant aged five months [in French]. Ann Pediatr (Paris) 1969;16:263–71. 4240638

[8] Van der Hauwaert LG Dumoulin M Moerman P Congenital atresia of left coronary ostium. Br Heart J 1982;48:298–300. 7104124 10.1136/hrt.48.3.298 PMC481246

[9] Gay F Vouhe P Lecompte Y Atresia of the left main coronary ostium surgical repair in a 2-month old infant [in French]. Arch Mal Coeur Vaiss 1989;82:807–10. 2525374

[10] Sato S Majima T Kawaguchi T Congenital atresia of the left main coronary artery ostium: a case suffering from ventricular tachycardia. Nihon Kyobu Geka Gakkai Zasshi 1990;38:1467–74. 2246532

[11] Fortune RL Baron PJ Fitzgerald JW Atresia of the left main coronary artery: repair with left internal mammary artery bypass. J Thorac Cardiovasc Surg 1987;94:150–1. 3496498

[12] Betrian Blasco P Girona Comas J Murtra Ferre M Angor in a child: left main coronary artery atresia. Int J Cardiol 2006;108:109–10. 16516702 10.1016/j.ijcard.2005.01.062

[13] Takeuchi D Mori Y Kishi K Percutaneous transluminal coronary angioplasty for postoperative left coronary artery stenosis following surgical reconstruction of congenital atresia of the left main coronary artery. Circ J 2009;73:2360–2. 19491509 10.1253/circj.cj-08-0136

[14] Zheng J Song H Jiang S Congenital atresia of the left main coronary artery with noncompaction of the ventricular myocardium in an asymptomatic young child. Pediatr Cardiol 2013;34:1998–2002. 23124386 10.1007/s00246-012-0545-8

[15] Xiao Y Han L Jin M Congenital atresia of left main coronary artery in 4 children: case report and literature review. Zhonghua Er Ke Za Zhi 2014;52:383–6. 24969939

[16] D'Souza TF Samuel BP Vettukattil JJ Surgical treatment of neonate with congenital left main coronary artery atresia. Ann Thorac Surg 2016;101:352–5. 26694277 10.1016/j.athoracsur.2014.12.104

[17] Bounds RL Kuebler J Cholette JM Left main coronary artery atresia in an infant with inclusion-cell disease. World J Pediatr Congenit Heart Surg 2018;9:246–50. 27619328 10.1177/2150135116664701

[18] Allen HD Moller JH Formanek A Atresia of the proximal left coronary artery associated with supravalvular aortic stenosis. Surgical treatment. J Thorac Cardiovasc Surg 1974;67:266–71. 4544028

[19] Rosenkranz ER Murphy DJ Cosgrove DM Surgical management of left coronary artery ostial atresia and supravalvular aortic stenosis. Ann Thorac Surg 1992;54:779–81. 1417243 10.1016/0003-4975(92)91031-4

[20] Rubio Alonso B Jurado Roman A Alonso Charterina S Left main coronary artery atresia in an asymptomatic elderly adult. Rev Esp Cardiol (Engl Ed) 2015;68:436. 25449815 10.1016/j.rec.2014.06.023

[21] Hung JW Ischemic (functional) mitral regurgitation. Cardiol Clin 2013;31:231–6. 23743075 10.1016/j.ccl.2013.04.003

[22] Leitz KH Oster H Keutel J Use of the left internal thoracic artery to correct a left main coronary atresia. J Thorac Cardiovasc Surg 1987;35:345–7. 10.1055/s-2007-1020260 2448902

[23] Dymond D Camm J Stone D Dual isotope stress testing in congenital atresia of left coronary ostium. Applications before and after surgical treatment. Br Heart J 1980;43:270–5. 7437174 10.1136/hrt.43.3.270 PMC482275

[24] Kaczorowski DJ Sathanandam S Ravishankar C Coronary ostioplasty for congenital atresia of the left main coronary artery ostium. Ann Thorac Surg 2012;94:1307–10. 22835554 10.1016/j.athoracsur.2012.04.072

[25] Byrum CJ Blackman MS Schneider B Congenital atresia of the left coronary ostium and hypoplasia of the left main coronary artery. Am Heart J 1980;99:354–8. 7355698 10.1016/0002-8703(80)90351-8

